# Single-molecule imaging analysis reveals the mechanism of a high-catalytic-activity mutant of chitinase A from *Serratia marcescens*

**DOI:** 10.1074/jbc.RA119.012078

**Published:** 2020-01-10

**Authors:** Akasit Visootsat, Akihiko Nakamura, Paul Vignon, Hiroki Watanabe, Takayuki Uchihashi, Ryota Iino

**Affiliations:** ‡ Department of Functional Molecular Science, School of Physical Sciences, Graduate University for Advanced Studies (SOKENDAI), Hayama, Kanagawa 240–0193, Japan; § Institute for Molecular Science, National Institutes of Natural Sciences, Okazaki, Aichi 444-8787, Japan; ¶ Chimie ParisTech, Paris 75231, France; ‖ Department of Physics, Nagoya University, Nagoya, Aichi 464–8601, Japan; ** Exploratory Research Center on Life and Living Systems (ExCELLS), National Institute of Natural Science, Okazaki, Aichi 444–8787, Japan

**Keywords:** chitinase, processivity, single-molecule biophysics, Serratia marcescens, chitin, biomass conversion, biotechnology, chitin degradation, high-speed atomic force microscopy, single-molecule fluorescence imaging

## Abstract

Chitin degradation is important for biomass conversion and has potential applications for agriculture, biotechnology, and the pharmaceutical industry. Chitinase A from the Gram-negative bacterium *Serratia marcescens* (*Sm*ChiA) is a processive enzyme that hydrolyzes crystalline chitin as it moves linearly along the substrate surface. In a previous study, the catalytic activity of *Sm*ChiA against crystalline chitin was found to increase after the tryptophan substitution of two phenylalanine residues (F232W and F396W), located at the entrance and exit of the substrate binding cleft of the catalytic domain, respectively. However, the mechanism underlying this high catalytic activity remains elusive. In this study, single-molecule fluorescence imaging and high-speed atomic force microscopy were applied to understand the mechanism of this high-catalytic-activity mutant. A reaction scheme including processive catalysis was used to reproduce the properties of *Sm*ChiA WT and F232W/F396W, in which all of the kinetic parameters were experimentally determined. High activity of F232W/F396W mutant was caused by a high processivity and a low dissociation rate constant after productive binding. The turnover numbers for both WT and F232W/F396W, determined by the biochemical analysis, were well-replicated using the kinetic parameters obtained from single-molecule imaging analysis, indicating the validity of the reaction scheme. Furthermore, alignment of amino acid sequences of 258 *Sm*ChiA-like proteins revealed that tryptophan, not phenylalanine, is the predominant amino acid at the corresponding positions (Phe-232 and Phe-396 for *Sm*ChiA). Our study will be helpful for understanding the kinetic mechanisms and further improvement of crystalline chitin hydrolytic activity of *Sm*ChiA mutants.

## Introduction

Chitin is one of the most abundant polysaccharides found in nature, only secondary to cellulose. Chitin is a water-insoluble polysaccharide consisting of β-1,4 glycosidic linkages of the monomer GlcNAc, and it is mostly found in the shells of crustaceans, the exoskeleton of insects, and the cell walls of some fungi (1–3). Chitin degradation is important not only in nature, but also in industrial applications such as biomass conversion, and has potential applications in agriculture, biotechnology, and the pharmaceutical industry (4, 5). Due to its stable crystalline structure, chitin is very durable, only decomposing at very high temperatures and under high pressure (6).

**Figure 1.**
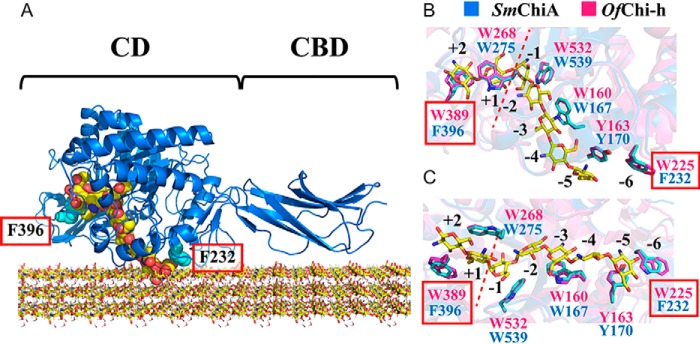
**Schematic model of *Sm*ChiA.** *A*, crystal structure of *Sm*ChiA complexed with (GlcNAc)_7_ (*sphere model*) and crystalline chitin (*stick model*). The *schematic* shows the CD and CBD. Phe-232 and Phe-396 (*sphere model*, *colored* with *cyan*) are located at the entrance and exit of the chitin-binding cleft, respectively. *B* and *C*, structural superimposition to compare the aromatic residues inside the chitin-binding cleft of *Sm*ChiA (*blue* and side chain highlighted with *cyan*; PDB entry 1CTN) and *Of*Chi-h bound with (GlcNAc)_7_ (*pink*; PDB entry 5GQB) in *side view* (*B*) and *bottom view* (*C*). The *red boxes* indicate the two different aromatic residues in *Sm*ChiA and *Of*Chi-h. The *numbers* (−6 to +2) represent the chitin-binding subsites of *Sm*ChiA according to the standard nomenclature (38, 39). The *red dashed lines* in *B* and *C* show the position of the bond cleavage.

There are many naturally occurring organisms that possess the ability to degrade chitin under mild conditions by using enzymes called chitinases (7). According to the Carbohydrate-Active enZYmes database (CAZY: http://www.cazy.org/)^2^ (52), chitinase is classified in the glycoside hydrolase family 18 (EC 3.2.1.14) and functions to catalyze the hydrolysis of the β-1,4 glycosidic bonds of chitin. This hydrolysis occurs on the crystalline surface of chitin. Moreover, the chitinases of the bacterium *Serratia marcescens* are a well-known model for the study of chitin degradation. *S. marcescens* chitinase A (*Sm*ChiA),^3^ an exo-processive enzyme, is the most powerful enzyme among the chitinases of *Serratia* in the hydrolysis of crystalline chitin (8, 9). *Sm*ChiA is a linear molecular motor enzyme that hydrolyzes chitin from the reducing end and works in the extracellular environment without the need for ATP. *Sm*ChiA is composed of two domains: a catalytic domain (CD) and a chitin-binding domain (CBD) (Fig. 1*A*) (10). Both the chitin-binding cleft of CD and the chitin-binding surface of CBD have aromatic residues lined along them. These aromatic residues play important roles in both substrate binding and the hydrolytic activity and processivity of the *Sm*ChiA (11, 12).

Processive enzymes play the important roles in various biological activities such as DNA/RNA synthesis (13, 14), cargo transport (15, 16), and protein (17, 18) and polysaccharide (19–21) degradations. Once processive enzymes bind to their substrates, they can repeat multiple cycles of catalysis without dissociation (22, 23). Processive cellulases and chitinases are well-known examples of the processive enzymes that perform multiple rounds of hydrolytic cleavage of cellulose and chitin, respectively (19–21). Processivity prevents the dissociation/reassociation process once the enzyme binds to the end of a single polymer chain, thereby reducing the number of times the enzyme rebinds to the end of the same substrate chain (20, 24). Processive cellulases and chitinases share the similar feature of a long and deep substrate-binding cleft and substrate-binding surface, which contain aromatic amino acid residues (20, 21, 25). These aromatic residues play an important role in the carbohydrate-protein interaction by which hydrophobic stacking (CH-π interaction) is formed between the aromatic side chain and sugar ring. This interaction is thought to be beneficial for processivity by reducing the sliding energy of the polymer carbohydrate chain (20, 26–29).

Studies on processivity of the cellulases and chitinases using biochemical methods, such as the fluorescence labeling of the substrate (30, 31) and ^14^C-labeled chitin (32), or via the use of biosensors (33) have been performed extensively. However, they often involve complicated procedures and have some limitations. Furthermore, processivity cannot be directly measured using a biochemical assay as it requires interpretations and is usually estimated from the dissociation rate. Recently, single-molecule imaging methods with fluorescence microscopy or high-speed atomic force microscopy (HS-AFM) have been used to directly visualize the processive movement of the enzymes because they are more straightforward than biochemical methods (34–37). In our previous study (37), we reported not only the processivity but also the kinetic parameters of *Sm*ChiA WT (His_6_-tagged), including the binding rate constant (*k*_on_), dissociation rate constant (*k*_off_), translational velocity (*k*_tr_), and productive binding ratio, obtained using single-molecule fluorescence imaging.

Recently, Liu *et al.* (38) reported the structure alignment of the substrate-binding cleft of *Ostrinia furnacalis* chitinase-h (*Of*Chi-h) and *Sm*ChiA. As a result, two different aromatic residues, Phe-232 and Phe-396 for *Sm*ChiA and Trp-225 and Trp-389 for *Of*Chi-h, were found in the beginning and end of the cleft (at the chitin-binding subsites −6 and +2) (38, 39), respectively, as shown in Fig. 1 (*B* and *C*). In addition, the mutation of these two positions of *Sm*ChiA into those of *Of*Chi-h (F232W/F396W) showed a higher hydrolytic activity against crystalline chitin compared with the *Sm*ChiA WT. However, the details of the mechanism for improved activity have not yet been reported. In the present study, we performed detailed biochemical analysis and single-molecule imaging analysis using fluorescence microscopy and HS-AFM to understand the mechanism by which F232W/F396W mutant showed a higher catalytic activity than WT. A reaction scheme including processive catalysis was used to explain the properties of *Sm*ChiA WT and F232W/F396W, in which all the kinetic parameters were experimentally determined. We also performed amino acid sequence alignment of 258 *Sm*ChiA-like proteins and revealed the predominant aromatic amino acid residues responsible for the chitin binding.

## Results

### SmChiA F232W/F396W mutant showed higher k_cat_ than WT

The previous study by Liu *et al.* (38) compared the hydrolytic activity of *Of*Chi-h, *Sm*ChiA WT, and *Sm*ChiA F232W/F396W. They found that the *Sm*ChiA F232W/F396W mutant showed higher hydrolytic activities against several insoluble chitin substrates than *Sm*ChiA WT; however, these results were obtained at only one substrate concentration (38). In our previous study (37), we used a range of crystalline chitin concentrations (0–2 mg/ml) to estimate the *k*_cat_ and *K_m_* values of *Sm*ChiA WT. In the present study, to confirm the high catalytic activity of the F232W/F396W mutant and estimate *k*_cat_ and *K_m_*, we measured the hydrolytic activity of *Sm*ChiA WT and F232W/F396W at various crystalline chitin concentrations (0–6 mg/ml) using a liquid-handling robot, developed in our previous report (40). The measurement was performed in two independent experiments carried out in triplicate. It is worth noting that contrary to the previous study (37), in the present study, His_6_ tags were removed from the C terminus of the constructs during purification. This was done as positive charges of His_6_ may change the binding/dissociation dynamics of *Sm*ChiA against the crystalline chitin immobilized on the surface of a negatively charged glass surface. Furthermore, fluorescently labeled enzymes (Cy3-D415C WT and F232W/F396W) were used for all biochemical measurements as these enzymes were also used for the single-molecule imaging analysis described later. Note that in our previous study (37), we confirmed that there is no significant difference between hydrolytic activities of Cy3-labeled and unlabeled enzymes.

**Figure 2.**
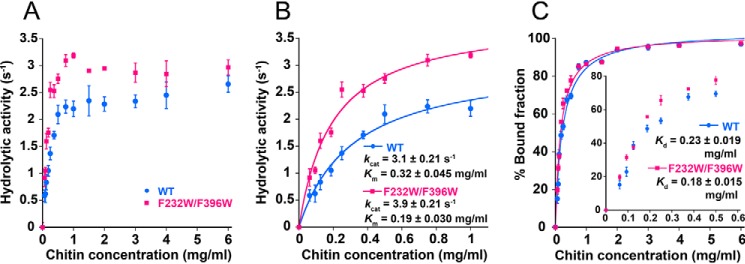
**Biochemical analysis.** *A*, hydrolytic activity of *Sm*ChiA WT (*blue*) and F232W/F396W (*pink*) at various concentrations of crystalline chitin (0–6 mg/ml). *B*, same as *A* at a low concentration range (0–1 mg/ml). The data points were fitted with the Michaelis–Menten equation to estimate *k*_cat_ and *K_m_* of WT and F232W/F396W. Hydrolytic activity was measured in 50 mm sodium phosphate (pH 6.0) at 25 °C. *C*, the bound fraction of WT and F232W/F396W at various concentrations of crystalline chitin (0–6 mg/ml). *Inset*, the low chitin concentration range (<0.6 mg/ml). The amount of free enzymes was used to calculate the bound fraction percentage. The distribution was fitted with the Langmuir equation to estimate the dissociation constant (*K_d_*). *Error bars*, S.D. of the sextupled (*A* and *B*) or triplicate (*C*) measurements.

As a result, F232W/F396W was found to show higher hydrolytic activity than WT in all of the crystalline chitin concentrations tested (Fig. 2*A*). At a high crystalline chitin concentration (defined as more than 1 mg/ml), the hydrolytic activities of WT and F232W/F396W tended to show a degree of inhibition and were not appropriate within the Michaelis–Menten equation. Thus, we used the hydrolytic activity at a low chitin concentration range (0–1 mg/ml) to ensure fit within the Michaelis–Menten equation (Fig. 2*B*). The turnover number (*k*_cat_) and Michaelis constant (*K_m_*) for WT and F232W/F396W on crystalline chitin were subsequently estimated to be 3.1 ± 0.21 and 3.9 ± 0.21 s^−1^ and 0.32 ± 0.045 and 0.19 ± 0.030 mg/ml, respectively (Table 1). Furthermore, the values of *k*_cat_/*K_m_* for WT and F232W/F396W were 10 and 21 ml mg^−1^ s^−1^, respectively. This result suggests a 2 times larger rate constant of productive binding for F232W/F396W than that for WT.

**Table 1:** **Summary of kinetic parameters determined by biochemical analysis**

Kinetic parameters	*k*_cat_*^a^*	*K_m_^a^*	*k*_cat_/*K_m_*	*K_d_^b^*
	*s*^−*1*^	*mg/ml*	*ml mg*^−*1*^ *s*^−*1*^	*mg/ml*
WT	3.1 ± 0.21	0.32 ± 0.045	10	0.23 ± 0.019
F232W/F396W	3.9 ± 0.21	0.19 ± 0.030	21	0.18 ± 0.015

*^a^ k*_cat_ and *K_m_* were estimated from the biochemical activity measurement at a low chitin concentration range (0–1 mg/ml) with the fitting by the Michaelis–Menten equation.

*^b^ K_d_* was estimated from the bound fraction analysis with the fitting by Langmuir's equation.

In addition, we performed a biochemical binding assay to compare the ratio of bound fractions between WT and F232W/F396W at various crystalline chitin concentrations. The free enzymes in the solution were used to calculate the percentage of the bound fraction. The plot was fitted using Langmuir's equation to estimate the dissociation constant (*K_d_*) (Fig. 2*C*). At a crystalline chitin concentration below 0.6 mg/ml, the binding affinity of F232W/F396W was found to be slightly higher than that of the WT (Fig. 2*C*, *inset*). At a concentration of 2 mg/ml crystalline chitin, almost all of the enzymes of both the WT and F232W/F396W were found to be bound to the crystalline chitin surface (over 90%). The values of *K_d_* for WT and F232W/F396W were 0.23 ± 0.019 and 0.18 ± 0.015 mg/ml, respectively (Table 1). These results indicate that the binding affinity increased slightly as a result of the mutation of two phenylalanine residues into tryptophan residues.

### No significant differences in binding and dissociation rate constants and productive binding ratio for WT and F232W/F396W

**Figure 3.**
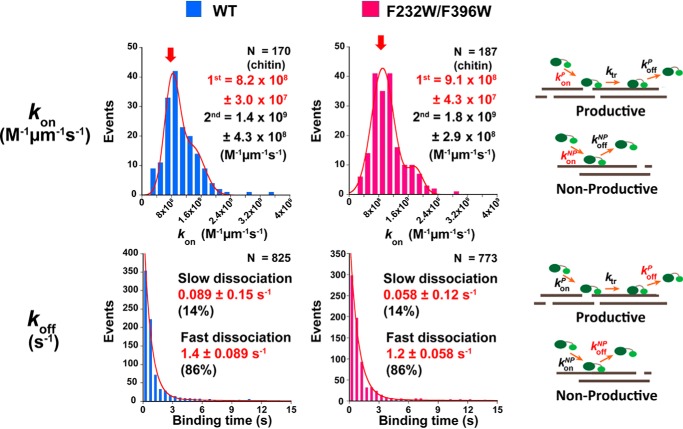
**Comparison of the distribution of *k*_on_ and *k*_off_ of *Sm*ChiA WT (*blue*) and F232W/F396W (*pink*) obtained by single-molecule fluorescence imaging analysis.** *Top*, the distributions of *k*_on_ were fitted with the double Gaussian function. The first peak corresponds to the *k*_on_ against the single crystalline chitin microfibril. The sample number (*N*) in the distribution of *k*_on_ represents the number of crystalline chitins observed. *Bottom*, the distributions of *k*_off_ were fitted with the double-exponential decay function. A slow dissociation is associated with the binding of the enzyme to the hydrophobic crystalline chitin surfaces, whereas the fast dissociation is associated with the binding of the enzyme to the hydrophilic crystalline chitin surfaces. The sample number (*N*) in the distribution of *k*_off_ represents the number of *Sm*ChiA molecules observed.

To further clarify the mechanism responsible for the higher hydrolytic activity in the F232W/F396W mutant compared with the WT, we first performed single-molecule fluorescence imaging according to the methods described in our previous study (37). Note that in the single-molecule fluorescence imaging and HS-AFM observation, it is difficult to define the chitin concentrations because the chitin microfibrils are attached on the glass or mica surface. Both the *k*_on_ and *k*_off_ of the WT and F232W/F396W were found to be similar (Fig. 3). The distributions of *k*_on_ were fitted using the double Gaussian function (Fig. 3, *top*). The values of the peak positions for the WT were 8.2 ± 0.30 × 10^8^ and 1.4 ± 0.43 × 10^9^ m^−1^ μm^−1^ s^−1^, whereas those of the F232W/F396W were 9.1 ± 0.43 × 10^8^ and 1.8 ± 0.29 × 10^9^ m^−1^ μm^−1^ s^−1^, respectively. The multiple peaks of *k*_on_ were related to the bundles of chitin microfibrils, wherein the first peak was represented by *k*_on_ for a single crystalline chitin microfibril, as explained in our previous study (37). Essentially, no significant differences in overall *k*_on_ between WT and F232W/F396W were obtained (Table 2).

**Table 2:** **Summary of kinetic parameters determined by single-molecule analysis and reaction scheme**

Kinetic parameters	*k*_on_*^a^*	*k*_off_^NP^*^a,b^*	*k*_on_^P^/*k*_on_^NP^*^a,c^*	*k*_tr_*^d^*	*k*_pc_*^e^*	*P^d,f^*	*k*_off_^P^*^d^*	*k*_cat_*^g^*
	*m*^−*1*^ μ*m*^−*1*^ *s*^−*1*^	*s*^−*1*^	*n^P^*/*n^NP^*	*nm/s*	*s*^−*1*^		*s*^−*1*^	*s*^−*1*^
WT	8.2 × 10^8^ ± 3.0 × 10^7^	1.2	0.074 ± 0.0041	52 ± 0.53	50	30	1.4 ± 0.26	2.9
F232W/F396W	9.1 × 10^8^ ± 4.3 × 10^7^	0.99	0.076 ± 0.0089	51 ± 0.72	49	52	0.82 ± 0.089	4.1

*^a^ k*_on_, *k*_on_^P^/*k*_on_^NP^, and *k*_off_^NP^ values were obtained from single-molecule fluorescence imaging analysis.

*^b^ k*_off_^NP^, which was the average *k*_off_, was assumed to only contain nonproductive binding.

*^c^ k*_on_^P^/*k*_on_^NP^ or *n*^P^/*n*^NP^ was denoted as the productive binding ratio, where *n*^P^ and *n*^NP^ represent the number of moving and non-moving molecules for a total of 40 s observation, respectively. For the analysis, we defined moving molecules as the molecules which showed movements larger than 20 nm for 3 or more frames.

*^d^ k*_tr_, processivity (*P*), and *k*_off_^P^ were obtained by HS-AFM observation. The molecules that showed movements for 3 or more frames were analyzed.

*^e^ k*_tr_ was used to denote the translational velocity. *k*_pc_ was calculated by dividing the *k*_tr_ value by the step size (a product size, 1.04 nm).

*^f^ P* was estimated from the run length divided by the step size (a product size, 1.04 nm).

*^g^ k*_cat_ was calculated from Equation 3.

The distribution of *k*_off_ was fitted using a double-exponential decay function (Fig. 3, *bottom*). The slow and fast dissociations were associated with the bindings of the *Sm*ChiA to the hydrophobic and hydrophilic surfaces of crystalline chitin, respectively (37). The ratios of the slow and fast dissociations were calculated from the area under the fitting curves. The slow dissociation rate constants for WT and F232W/F396W were 0.089 ± 0.15 s^−1^ (14%) and 0.058 ± 0.12 s^−1^ (15%), respectively. The fast dissociation rate constants for WT and F232W/F396W were 1.4 ± 0.089 s^−1^ (86%) and 1.2 ± 0.058 s^−1^ (85%), respectively. Subsequently, the average value of *k*_off_ was calculated from summation of the *k*_off_ and the ratio of the slow dissociation fraction and that of the fast dissociation fraction. As a result, the average values of *k*_off_ for WT and F232W/F396W were 1.2 and 0.99 s^−1^, respectively (Table 2). As described below, we used these values of *k*_off_ as those of *k*_off_ for nonproductive binding (*k*_off_^NP^).

Next, we measured the productive binding ratio. Productive binding only occurs when *Sm*ChiA binds to the reducing end of the chitin chain on the hydrophobic surface of a crystalline chitin. Therefore, the productive binding ratio of *Sm*ChiA is low, as was determined by single-molecule imaging analysis in our previous study (37). In the present study, the productive binding ratio was determined from the ratio of the number of moving molecules (*n*^P^) and nonmoving molecules (*n*^NP^) after the binding to the chitin surface. For measurement, we further improved the localization precision of single-molecule fluorescence imaging to identify the slowly moving molecules more precisely (4.3 and 3.8 nm in the *x* and *y* direction at 3 frames per second (fps), with a laser at 1 μW/μm^2^ power). The productive binding ratios for WT and F232W/F396W were 0.074 ± 0.0041 and 0.076 ± 0.0089, respectively, and approximately the same (Table 2).

### F232W/F396W showed high processivity and low dissociation rate after productive binding

**Figure 4.**
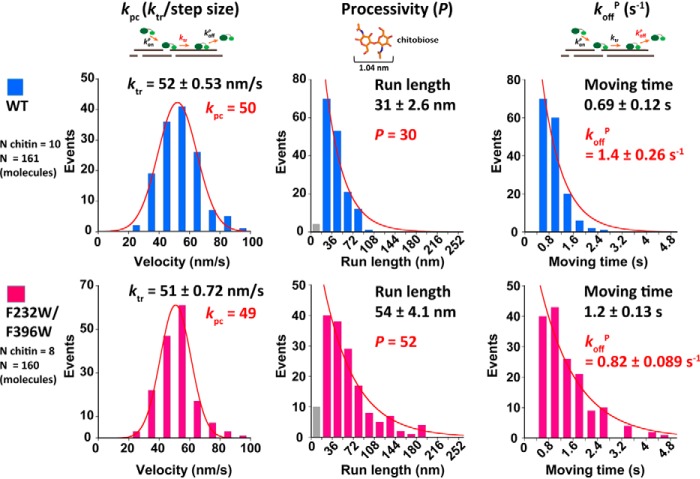
**Comparison of the distribution of *k*_tr_, run length, and moving time of WT (*blue*) and F232W/F396W (*pink*) obtained by HS-AFM.** *Left*, the distributions of *k*_tr_ were fitted with the single Gaussian function. The processive catalysis rate constant (*k*_pc_) was calculated by dividing *k*_tr_ with the step size (product size: 1.04 nm). *Center* and *right*, the distributions of run length and moving time were fitted with the single-exponential decay function. The processivity (*P*) was estimated by dividing the run length by the step size. The inverse of the moving time was determined as the productive dissociation rate constant (*k*_off_^P^), because all of the molecules analyzed by HS-AFM were moving molecules. The N and N chitin represent the number of *Sm*ChiA molecules and chitin microfibrils, respectively. The first bins (*gray bars*) of the run length were not included for fitting, because precise measurement of short run length was difficult.

As no significant difference was found between the WT and F232W/F396W using single-molecule fluorescence imaging analysis, we then applied single-molecule imaging with HS-AFM to improve the localization precision. Several chitin microfibrils were observed to avoid heterogeneity on the crystalline chitin surface. At least 10 molecules per chitin were observed in order to estimate the translational velocity (*k*_tr_), run length, and moving time (Fig. 4). The distributions of *k*_tr_ were fitted with the single Gaussian function (Fig. 4, *left*). As a result, the WT and F232W/F396W were found to have a similar *k*_tr_, 52 ± 0.53 and 51 ± 0.72 nm/s, respectively. Then the processive catalysis rate constant (*k*_pc_) was calculated from the *k*_tr_ divided by the step size 1.04 nm, which is same as the size of the reaction product, chitobiose (36). The values of *k*_pc_ for WT and F232W/F396W were 50 and 49, respectively. The distributions of run length (Fig. 4, *center*) and moving time (Fig. 4, *right*) were fitted with the single-exponential decay function. Note that the first bins (*gray bars*) of the run length were not included for fitting, because precise measurement of short run length was difficult. The values of run length for WT and F232W/F396W were 31 ± 2.6 and 54 ± 4.1 nm, respectively, and F232W/F396W showed a longer run length. Then the values of processivity (*P*, run length divided by step size) were calculated as 30 and 52 for WT and F232W/F396W, respectively. Because F232W/F396W showed longer run length than WT, its moving time was also longer. The values of moving time for WT and F232W/F396W were 0.69 ± 0.12 and 1.2 ± 0.13 s, respectively. Because we only analyzed moving molecules in the HS-AFM observation, the inverse of the moving time corresponds to the productive dissociation rate constant, *k*_off_^P^. The values of *k*_off_^P^ for WT and F232W/F396W were 1.4 ± 0.26 and 0.82 ± 0.089 s^−1^, respectively (Table 2).

### Reaction scheme and calculation of k_cat_ from kinetic parameters obtained by single-molecule analysis

**Figure 5.**
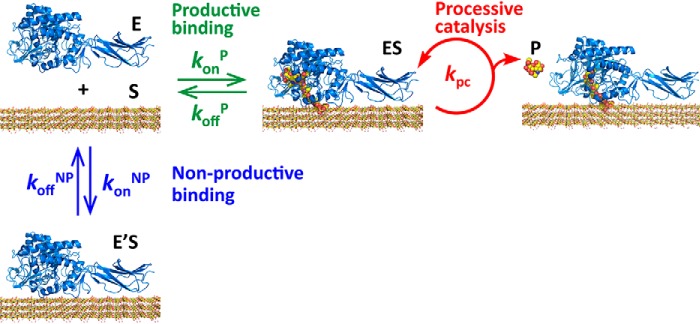
**Reaction scheme of the processive catalysis of *Sm*ChiA described with the kinetic parameters obtained experimentally.** The *green*, *blue*, and *red arrows* denote the productive binding/dissociation, nonproductive binding/dissociation, and processive catalysis (hydrolysis cycle), respectively. *E*, enzyme (*Sm*ChiA); *S*, substrate (crystalline chitin); *ES*, enzyme-substrate complex after productive binding; *E*′*S*, enzyme-substrate complex after nonproductive binding; *P*, product (chitobiose).

To calculate the *k*_cat_ from the kinetic parameters determined by single-molecule imaging analysis, a reaction scheme of *Sm*ChiA, including productive binding, nonproductive binding, and processive catalysis, is considered (Fig. 5). As also described above, *Sm*ChiA has two binding modes: productive and nonproductive bindings. In the reaction scheme shown in Fig. 5, these two modes are represented by four rate constants, the productive binding rate constant (*k*_on_^P^), the productive dissociation rate constant (*k*_off_^P^), the nonproductive binding rate constant (*k*_on_^NP^), and the nonproductive dissociation rate constant (*k*_off_^NP^). Furthermore, because *Sm*ChiA is a processive enzyme, another kinetic parameter is included, *k*_pc_. From this reaction scheme, we can derive the following equations to estimate *k*_cat_ and *K_m_*.

(Eq. 1) $$k_{\text{cat}}=\frac{k_{\text{on}}^{\text{P}}\cdot k_{\text{off}}^{\text{NP}}\cdot k_{\text{pc}}}{k_{\text{on}}^{\text{P}}\cdot k_{\text{off}}^{\text{NP}}+k_{\text{on}}^{\text{NP}}\cdot k_{\text{off}}^{\text{P}}}$$

(Eq. 2) $$K_{m}=\frac{k_{\text{off}}^{\text{P}}\cdot k_{\text{off}}^{\text{NP}}}{k_{\text{on}}^{\text{P}}\cdot k_{\text{off}}^{\text{NP}}+k_{\text{on}}^{\text{NP}}\cdot k_{\text{off}}^{\text{P}}}$$

As described, the values of *k*_off_^P^, *k*_off_^NP^, and *k*_pc_ for WT and F232W/F396W in Equation 1 were already determined by the single-molecule imaging analysis. Because the ratio of productive binding was very low in the single-molecule fluorescence imaging, we approximated the values of *k*_off_^NP^ with those of *k*_off_ (Fig. 3 and Table 2). The values of *k*_off_^P^ were obtained as the inverse of the moving time in the HS-AFM observation (Fig. 4 and Table 2). The values of *k*_pc_ were also estimated from the *k*_tr_ divided by the step size (1.04 nm) in the HS-AFM observation (Fig. 4 and Table 2).

On the other hand, quantitative determinations of absolute values of *k*_on_^P^ and *k*_on_^NP^ were difficult even with single-molecule imaging analysis. The values of *k*_on_ that were obtained from the single-molecule fluorescence imaging included both productive and nonproductive bindings. We could not distinguish productive and nonproductive bindings because of low excitation laser power (0.14 μW/μm^2^) and low localization precision (8.6 and 8.4 nm in *x* and *y* directions, respectively) under the experimental conditions used in the *k*_on_ and *k*_off_ measurements. Therefore, to calculate the values of *k*_cat_ from the kinetic parameters obtained, we modified Equation 1 and used the ratio of *k*_on_^P^ and *k*_on_^NP^ as follows.

(Eq. 3) $$k_{\text{cat}}=\frac{\frac{k_{\text{on}}^{\text{P}}}{k_{\text{on}}^{\text{NP}}}\cdot k_{\text{off}}^{\text{NP}}\cdot k_{\text{pc}}}{\frac{k_{\text{on}}^{\text{P}}}{k_{\text{on}}^{\text{NP}}}\cdot k_{\text{off}}^{\text{NP}}\cdot k_{\text{off}}^{\text{P}}}$$

The ratio of *k*_on_^P^ and *k*_on_^NP^ in Equation 3 corresponds to the productive binding ratio (*n*^P^/*n*^NP^) (Table 2), determined from the ratio of *n*^P^ and *n*^NP^ in the single-molecule fluorescence imaging with high excitation laser power (1 μW/μm^2^) and high localization precision (∼4 nm for both *x* and *y* directions). The obtained values of *k*_cat_ for WT and F232W/F396W were 2.9 and 4.1 s^−1^, respectively (Table 2). These values showed good agreement with those determined by the biochemical assay, which were 3.1 and 3.9 s^−1^ for WT and F232W/F396W, respectively (Table 1).

## Discussion

Our biochemical analysis confirmed that the F232W/F396W mutant of *Sm*ChiA has a higher hydrolytic activity than the WT, as shown previously (38) (Fig. 2). We found that F232W/F396W showed higher hydrolytic activity than WT at all chitin concentrations ranging from 0.063 to 6 mg/ml. Hydrolytic activities were slightly inhibited for both WT and F232W/F396W at chitin concentrations higher than 1 mg/ml and could not be fitted using the Michaelis–Menten equation (Fig. 2*A*). This inhibition can be either the product or substrate inhibition, and additional experiments are required to understand the mechanism.

At low chitin concentration range (0–1 mg/ml), the Michaelis–Menten equation could be applied to obtain an estimate of the *k*_cat_ and *K_m_* (Fig. 2*B*). The *k*_cat_ value for F232W/F396W (3.9 s^−1^) was higher than that for WT (3.1 s^−1^) (Table 1). The *K_m_* value for F232W/F396W (0.19 mg/ml) was lower than for WT (0.32 mg/ml), indicating that the affinity of the productive binding to the crystalline chitin of this mutant was higher than that of the WT. Actually, the F232W/F396W slightly increased the binding affinity to crystalline chitin, including productive and nonproductive bindings, as demonstrated by the results of biochemical bound fraction analysis, especially at low chitin concentrations (Fig. 2*C*). The *K_d_* values for WT and F232W/F396W were consistent with the *K_m_* values (Table 2). Because Phe was mutated to Trp at two positions, the surface area that engages the CH-π interactions and the electronegativity of the π-system can be increased (41).

Among the various kinetic parameters analyzed, processivity was the only one parameter for which F232W/F396W showed a higher value than the WT. The processivity of WT obtained by HS-AFM using β-chitin as a substrate was almost identical with that estimated in a previous report using HS-AFM (34) (values of 30 and 29 for the present and previous studies, respectively) (Fig. 4). Moreover, the processivity of WT was also similar to the apparent processivity (36 ± 5) estimated by biochemical analysis using α-chitin (32).

**Figure 6.**
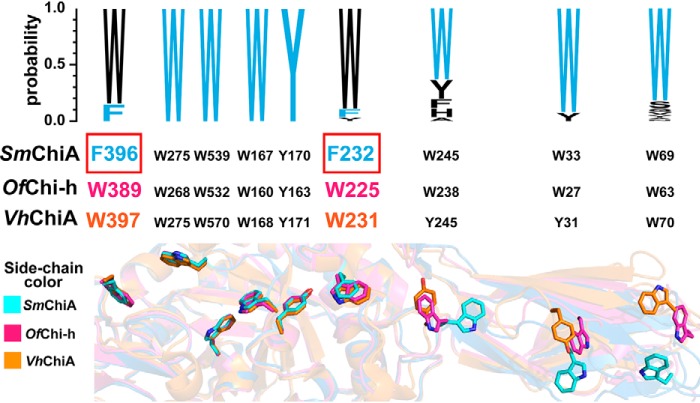
**Comparison of the aromatic amino acid residues responsible for the binding to the crystalline chitin surface and chain sliding into the chitin binding cleft.** The amino acid sequences of 258 *Sm*ChiA-like proteins were aligned and visualized by Clustal-Omega and WebLogo3. The aromatic amino acids of *Sm*ChiA are shown in *cyan*. The alignment shows the conservation of several aromatic amino acid residues involved in chitin binding. The structural alignment image was constructed by superimposing the crystal structure of *Sm*ChiA (PDB entry 1CTN; *blue* and side chain *highlighted* with *cyan*), *Of*Chi-h (PDB entry 5GQB; *pink*), and *Vh*ChiA (PDB entry 3B8S; *orange*) using PyMOL software. The side chains of aromatic amino acid residues are shown as *stick models*.

For Phe-232 and Phe-396 of *Sm*ChiA, single point mutations have been reported previously. The mutation of Phe-232 to Ala dramatically decreased the hydrolytic activity. However, the binding affinity of F232A and WT against crystalline chitin were similar (12). In addition, the Trp-231 of *Vibrio harveyi* chitinase A (*Vh*ChiA) corresponding to the Phe-232 in *Sm*ChiA (Fig. 6), was mutated to Phe (W231F), which resulted in nondetectable levels of activity against the crystalline chitin substrate (42). Phe-232 may be important in guiding the chitin chain into the substrate binding cleft not only in *Sm*ChiA but also in chitinase A in other organisms. Moreover, the mutation of F396A decreased the hydrolytic activity against the crystalline chitin. This F396A mutation increased the *K_d_* value 2-fold and slightly reduced the processivity compared with WT (11, 43). The slightly decreased *K_m_* was also observed when Phe-396 was mutated to Trp with D313N (44, 45). Taken together, F232W/F396W mutation in *Sm*ChiA would be better in guiding the chitin chain, whereby the binding affinity was increased and the degree of processivity was improved as a result of the larger aromatic surface area of Trp compared with Phe.

In previous studies, the hydrolytic activity of *Sm*ChiA against crystalline chitin was found to decrease after the introduction of an alanine mutation into the aromatic residues inside the substrate binding cleft (W167A, W275A, or F396A). These mutants showed reduced levels of processivity on the crystalline chitin (11, 32). On the other hand, interestingly, these mutants were found to outperform *Sm*ChiA WT in the hydrolysis of soluble chitin and soluble chitosan (11, 12). Furthermore, recently, the F232W/F396W *Sm*ChiA mutant was found to have an increased hydrolytic activity for insoluble chitin (insect cuticle, α-chitin, and chitin nanowhisker), but a decreased activity in ethylene glycol chitin, which is a water-soluble chitin substrate (38). In the present study, we found that high hydrolytic activity of the F232W/F396W against crystalline chitin resulted from high processivity. Our results indicate that processivity is an important factor for the hydrolysis of crystalline substrates. On the other hand, it remains elusive why the *Sm*ChiA alanine mutants (W167A, W275A, or F396A) and F232W/F396W, respectively, showed higher and lower hydrolytic activities than the *Sm*ChiA WT against the soluble substrates. To understand the mechanism and correlation with the processivity, measurements of the processivity of *Sm*ChiA WT and these mutants against the soluble substrates will be required.

The processivity (*P*) of *Sm*ChiA is related to *k*_off_^P^ (Fig. 4). If the value of *P* is high, that of *k*_off_^P^ is low (Table 2). Several studies have reported largely different values of the dissociation rate (30–33). The *k*_off_ from the chitin nanowhiskers was 0.012 ± 0.002 s^−1^, whereas the *k*_off_ from the α-chitin after 10 min and 2 h of incubation was 0.0028 ± 0.0003 and 0.0015 ± 0.0005 s^−1^, respectively (32). These values were much lower than those found in the present study and our previous study (37). Because the previous studies of processive cellulases reported different *k*_off_ values measured with different methods (31, 33, 46–51), this difference could also occur in the case of processive chitinase. For chitinase, biochemical analysis revealed that the *k*_off_ value is also dependent on the type of substrate and the reaction time (32). It is worth noting that in biochemical analysis, it is very difficult to distinguish productive and nonproductive bindings. On the other hand, in our single-molecule analysis, we have analyzed only productively bound *Sm*ChiA molecules moving on the surface of crystalline chitin.

To gain further insight into the aromatic amino acid residues in the substrate-binding cleft, we then performed bioinformatics analysis. The amino acid sequence of *Sm*ChiA and those of 258 *Sm*ChiA-like proteins obtained by the Blast analysis were aligned (Fig. 6). As a result, we found that Phe-232 and Phe-396 of *Sm*ChiA were not conserved, and the Trp was predominant and higher than 80% at both positions. Interestingly, Hudson *et al.* (41) previously reported that, for most proteins that interact with carbohydrates, aromatic side chains are involved, and the most preferable amino acid residue is tryptophan. Furthermore, we also found that not only are the aromatic residues within the substrate binding cleft, but also many other amino acid residues in the substrate binding cleft are not conserved in *Sm*ChiA (data not shown). Our results strongly suggest that although *Sm*ChiA is the most studied processive chitinase, the amino acid sequence is not optimized for high hydrolytic activity. Bioinformatics analysis used in this study will be helpful to find the amino acid residues that are not conserved in *Sm*ChiA, and these amino acid residues can be targets of the mutation for further improvement of the crystalline chitin hydrolytic activity. Furthermore, our single-molecule imaging analysis and reaction scheme will make it possible to understand the mechanisms of the highly active mutants.

To conclude, the F232W/F396W mutant of *Sm*ChiA showed high processivity and low productive dissociation rate constant, which resulted in a *k*_cat_ value higher than that for WT. The values of *k*_cat_ for F232W/F396W and WT obtained by biochemical analysis were well-reproduced by the kinetic parameters obtained by the single-molecule analysis, indicating the validity of the proposed reaction scheme. Our results highlight the importance of integration of biochemical analysis and single-molecule analysis to understand the mechanisms of *Sm*ChiA. To further clarify the mechanism in more detail, single-molecule analysis of the *Sm*ChiA mutants with higher time resolution and higher localization precision probed with a gold nanoparticle will be very important (36).

## Experimental procedures

### Preparation of enzymes

The *Sm*ChiA WT gene (including the D415C mutation for fluorescent labeling) (37) in the expression plasmid pET27b with the C-terminal Factor Xa (FaXa) recognition sequence and His_6_ tag was used as the template to introduce the F232W/F396W mutation by PCR. The PCR product was treated with 1 μl of DpnI (New England Biolabs) to reduce the template background at 37 °C for 15 min. After DpnI treatment, 1% agarose gel electrophoresis was performed. The target fragments were extracted and purified using a gel extraction kit (Promega). The purified DNA fragments were ligated using the NEBuilder Assembly Tool (New England Biolabs). The DNA fragments and reagent were mixed at a ratio of 1:1 (v/v) and then incubated at 50 °C for 30 min. After the ligation reaction, the samples were immediately used for transformation using *E. coli* (Tuner^TM^ DE3) as a host. Transformed cells were incubated for 1 h at 37 °C and spread on an LB plate with 25 μg/ml kanamycin. Single colonies were inoculated into 10 ml of LB medium with 25 μg/ml kanamycin and incubated overnight at 37 °C and 250 rpm. The plasmid containing mutant gene was purified from the harvested cell and the sequence was verified. The *Escherichia coli* colonies carrying *Sm*ChiA F232W/F396W or WT expression plasmids were cultured in 10 ml of LB medium with 25 μg/ml kanamycin at 37 °C and 250 rpm until *A*_600_ = 1. Then 5 ml of the culture was added to 1 liter of LB medium with 25 μg/ml kanamycin in a 3-liter flask and cultured at 37 °C and 130 rpm until *A*_600_ = 1.8. Then the media were cooled on ice water for 10 min, isopropyl β-d-1-thiogalactopyranoside was added at a final concentration of 500 μm, and cells were further cultured at 20 °C and 130 rpm overnight. The culture was then centrifuged at 6000 × *g* for 10 min. Ten times the volume of the cell weight of 50 mm sodium phosphate (pH 7.0), containing 100 mm NaCl, was added and supplemented with protease inhibitor mixture (cOmplete Mini, EDTA-free, Roche Applied Science). The cell suspension was sonicated on ice for 20 min at 3-s intervals. The disrupted cells were then centrifuged at 4 °C and 30,000 × *g* for 10 min. The supernatant was incubated with Ni-NTA Superflow (Qiagen) and equilibrated with 50 mm sodium phosphate (pH 7.0) containing 100 mm NaCl for 15 min at room temperature under gentle rotation. Then the Ni-NTA resin was packed into an open column and washed with 0 and 50 mm imidazole in 50 mm sodium phosphate (pH 7.0) containing 100 mm NaCl and eluted with 100 mm imidazole in 50 mm sodium phosphate (pH 7.0) containing 100 mm NaCl. The eluted fractions were pooled and concentrated to 500 μl using a 30,000 molecular weight cut-off VIVASPIN Turbo 50 (Sartorius). The sample was then injected into a Superdex 200 10/300 GL column (GE Healthcare) and eluted with 50 mm Tris-HCl (pH 8.0) containing 100 mm NaCl. The fractions were collected at a flow rate of 0.5 ml/min. The eluted fractions were mixed and concentrated to 200 μl using a 30-kDa molecular mass cut-off VIVASPIN Turbo 15 (Sartorius). Protein concentrations were estimated from the absorbance at 280 nm and the molar extinction coefficients (ϵ_280_ = 107,050 and 118,050 m^−1^ cm^−1^ for WT and F232W/F396W, respectively). The molar extinction coefficients were calculated by using the ProtParam in the Expasy bioinformatics resource portal web service (https://web.expasy.org/protparam/)^2^. One hundred microliters of 100 μm sample were incubated with 5 μl of 1 mg/ml FaXa protease (New England Biolabs) and 2 μl of 100 mm calcium chloride at 23 °C overnight to digest C-terminal His_6_. Then 10 μl of sodium phosphate (1 m, pH 7.0) was added to the FaXa-treated sample, and the sample was centrifuged at 4 °C and 16,000 × *g* for 10 min to precipitate calcium phosphate. The supernatant was applied to the Ni-NTA column to remove the cleaved His_6_ tag and undigested samples. The column was washed, and the flow-through fractions were collected with 50 mm sodium phosphate (pH 7.0) containing 100 mm NaCl. The collected fractions were mixed and concentrated to 500 μl using a 30-kDa molecular mass cut-off membrane (VIVASPIN Turbo 15, Sartorius). DTT at a final concentration of 10 mm was added to the sample to prevent the formation of disulfide bonds between D415C. The sample was then loaded onto a Superdex 200 10/300 GL column (GE Healthcare) with 50 mm sodium phosphate (pH 7.0) containing 100 mm NaCl to remove FaXa protease and DTT. Cy3-maleimide (GE Healthcare) was dissolved in DMSO and mixed with the sample at the same molar concentration as the enzyme before incubating for 1 h at room temperature. The unreacted Cy3-maleimide was removed using a NAP-5 column (GE Healthcare). The labeling ratio of Cy3 to enzyme (97% for WT and 83% for F232W/F396W mutant) was calculated from the absorbance at 280 and 550 nm, the molar extinction coefficient of the enzyme as described above, and the Cy3-maleimide (ϵ_280_ = 12,000 m^−1^ cm^−1^ and ϵ_550_ = 150,000 m^−1^ cm^−1^). The samples were then stored at −80 °C until further use. In this study, WT (D415C-Cy3) and F232W/F396W/D415C-Cy3 are described as WT and F232W/F396W, respectively.

Crystalline β-chitin was purified from Satsuma tube worms (*Lamellibrachia satsuma*), as described in a previous study (34).

### Biochemical measurement of chitinase hydrolytic activity

We used a liquid-handling robot, Beckman Coulter Biomek 4000, to measure the hydrolytic activity of all of the samples (blank, WT, and F232W/F396W). The samples were measured in triplicate simultaneously in 96-well plates. The purified enzymes were diluted to 100 nm using 100 mm sodium phosphate (pH 6.0) in a low-protein-binding microtube. In 96-well reaction plates, the diluted enzymes were incubated with crystalline chitin (0–6 mg/ml) at 25 °C for 30 min in a reaction mixture volume of 150 μl (1:1 (v/v) enzyme/substrate ratio) without shaking. The reactions were stopped with 200 μl of the Schales' reagent (500 mm sodium carbonate, 1.5 mm potassium ferricyanide). Insoluble chitin was separated on 96-well 1.2-μm hydrophilic low-protein-binding Durapore® membrane filter plates (Merck Millipore). The filtered solution was heated at 95 °C for 15 min, and 100 μl of the samples were transferred to 384-well clear plates. Absorbance at 420 nm was measured using a multimode microplate reader (SpectraMax iD3, Molecular Devices). The amounts of soluble products were calculated from the standard curve with chitobiose. The *error bars* shown in Fig. 2 (*A* and *B*) represent the S.D. values of the sextupled experiments.

### Biochemical bound fraction analysis

Bound fraction analysis was performed manually using a multichannel pipette. Each measurement condition was measured in triplicate. *Sm*ChiA WT and F232W/F396W were diluted to 100 nm using 100 mm sodium phosphate (pH 6.0) in a low-protein-binding microtube and transferred to a 96-well low-binding plate (Eppendorf). Then 100 nm enzyme was incubated with various concentrations of crystalline chitin (final concentration 0–6 mg/ml) at a 1:1 (v/v) enzyme/substrate ratio at 25 °C for 15 min. The 96-well plate was then centrifuged at 4400 rpm for 15 min at 25 °C. Then 100 μl of the supernatant (unbound fraction) was transferred to a 96-black well plate to measure the fluorescence. The fluorescence intensity was measured using a microplate reader (SpectraMax® iD3, Molecular Devices). The excitation and emission wavelengths were 550 and 610 nm, respectively, optimized by fluorescence spectral scanning. The intensities of the blank (no enzyme) were subtracted, and signal intensities were compared with that of the positive control (enzymes without chitin, 100% intensity). The percentages of the bound fractions were calculated, plotted, and fitted using Langmuir's equation. The *error bars* shown in Fig. 2*C* represent the S.D. values of the triplicate experiments.

### Single-molecule fluorescence imaging analysis

The coverslips used for the single-molecule fluorescence imaging were cleaned with ethanol and sonicated for 10 min before rinsing with MilliQ water and cleaned with 10 m potassium hydroxide overnight to remove any contaminants on the glass surface. Before using, the coverslips were rinsed with MilliQ water. Then 60 μl of 0.01 mg/ml crystalline chitin suspension was spin-coated on the coverslip before placement on the microscope stage. The observation area was determined using bright-field microscopy. Then 20 μl of 50 pm *Sm*ChiA WT or F232W/F396W in 50 mm sodium phosphate (pH 6.0) was dropped onto the coverslip. For *k*_on_ and *k*_off_ analysis, fluorescence images of single molecules were recorded at 4 fps at a laser power of 0.14 μW/μm^2^ with localization precisions of 8.6 and 8.4 nm in *x* and *y* directions, respectively. After observation, 10 μl of 10 nm WT was dropped onto the coverslip to strain the crystalline chitin.

The values of *k*_on_ were calculated from the number of *Sm*ChiA molecules bound to the single crystalline chitin divided by the *Sm*ChiA concentration, chitin length, and observation time (m^−1^ μm^−1^ s^−1^). Binding events were counted for 40 s after focusing. The length of the microfibrils was measured from the fluorescence images of crystalline chitin stained with 10 nm WT using ImageJ. The distributions of *k*_on_ were fitted using double Gaussian functions.

The binding time distribution was fitted with the double exponential decay functions, according to the following equation,

(Eq. 4) y=a·exp⁡(−bt)+c·exp⁡(−dt)

where *a*, *b*, *c*, and *d* are the fitting parameters.

The productive binding ratio was estimated from the ratio of the number of moving molecules and the number of nonmoving molecules in the initial 40-s movies after focusing. The observation condition was 1 μW/μm^2^ with 3 fps to improve the moving molecule–finding efficiency. The localization precisions in the *x* and *y* directions were 4.3 and 3.8 nm, respectively. For the analysis, we defined moving molecules as the molecules that showed movements larger than 20 nm (∼5 times larger than the localization precision) for 3 or more frames. Eight independent images were used to analyze and calculate the average value of the productive binding ratio. Before performing the detailed analysis of the image sequences, the trajectory of nonmoving molecules was verified to confirm whether there was any drift in the image sequences. If the image sequences showed any drift, they were not used for analysis.

### HS-AFM

HS-AFM observations were carried out using the system described previously (34), except the preparation of stage. The mica surface on the stage was freshly cleaved and coated with 2 μl of fluoro surf FS-1010S135-0.5 (Fluoro Technology) to make the surface hydrophobic and to have high affinity to the crystalline chitin. Then 10 μl of crystalline chitin suspension was dropped onto the surface before incubating at room temperature (∼25 °C) for 10 min and covered with a moisture cap. The surface was rinsed twice with 80 μl of 50 mm sodium phosphate (pH 6.0). Immobilized crystalline chitins were initially observed without the *Sm*ChiA in 78 μl of 50 mm sodium phosphate (pH 6.0). After that, 2 μl of chitinase was added to obtain an enzyme at a final concentration of 2 μm. The solution was then mixed gently, and the images were recorded at 5 fps.

Home-built software based on Igor Pro (WaveMetrics) was used for the visualization and analysis of the HS-AFM images. We analyzed moving molecules that showed movements for 3 or more frames. A linear tracking function was used to track the moving molecules. First, the region of interest was selected manually at the first and last frame of each individual moving molecule. The center of the region of interest was calculated using the software to obtain the position at each frame of tracking molecules. The translational velocity (*k*_tr_) of the moving molecules was calculated using the following equation,

(Eq. 5) $$k_{\text{tr}}=\frac{\sqrt{\Delta x^{2}\left({\text{nm}}^{2}\right)+\Delta y^{2}\left({\text{nm}}^{2}\right)}}{\text{moving}\;\text{time}\left(\text{s}\right)}$$

where Δ*x* and Δ*y* denote the difference between the start and the end positions of a moving molecule in the *x* and *y* direction, respectively.

The distributions of *k*_tr_ were fitted with the Gaussian function. The processive catalysis rate constant (*k*_pc_) was calculated by dividing *k*_tr_ by the step size of *Sm*ChiA (1.04 nm, the length of chitobiose). The distribution of the run length was fitted with the single-exponential decay function. The processivity was estimated from the obtained run length constant on the assumption that the *Sm*ChiA step size was 1.04 nm. The distribution of the moving time was fitted with the single-exponential decay function, and the inverse of the obtained moving time constant was used as the productive dissociation rate constant (*k*_off_^P^).

### Structure and sequence alignment by bioinformatics

The sequence of *Sm*ChiA and *Sm*ChiA-like proteins from the different organisms were obtained using the Protein Blast tool in the NCBI database (http://blast.ncbi.nlm.nih.gov). After the download of sequences, the signal sequences were removed according to the prediction of SignalP 5.0 (http://www.cbs.dtu.dk/services/SignalP)^2^ (53), with the appropriate organism group for prediction. The sequences for which SignalP could not predict the signal sequence were excluded from the alignment. The amino acid sequences of 258 chitinases were aligned and visualized using Clustal Omega (Clustalω: http://www.ebi.ac.uk/Tools/msa/clustalo)^2^ (54) and WebLogo (http://weblogo.threeplusone.com)^2^ (55), respectively. The structure of *Sm*ChiA (PDB entry 1CTN) was superimposed with *Of*Chi-h (PDB entry 5GQB) and *Vh*ChiA (PDB entry 3B8S) using the alignment function of PyMOL.

## Author contributions

A. V. data curation; A. V. and P. V. formal analysis; A. V., A. N., H. W., T. U., and R. I. methodology; A. V. writing-original draft; A. N. and R. I. conceptualization; A. N. resources; R. I. supervision; R. I. funding acquisition; R. I. project administration; R. I. writing-review and editing.

## Footnotes

2 Please note that the JBC is not responsible for the long-term archiving and maintenance of this site or any other third party hosted site.

## Footnotes

3 The abbreviations used are:

*Sm*ChiA: *Serratia marcescens* chitinase A
CBD: chitin-binding domain
CD: catalytic domain
FaXa: Factor Xa
HS-AFM: high-speed atomic force microscopy
*k*_off_^NP^: non-productive dissociation rate constant
*k*_off_^P^: productive dissociation rate constant
*k*_on_^NP^: non-productive binding rate constant
*k*_on_^P^: productive binding rate constant
*k*_pc_: processive catalysis rate constant
*k*_tr_: translational rate (velocity)
*Of*chi-h: *Ostrinia furnacalis* chitinase-h
*Vh*ChiA: *Vibrio harveyi* chitinase A
μW: microwatt
fps: frames per second
LB: Luria broth
Ni-NTA: nitrilotriacetic acid
PDB: Protein Data Bank.
